# The metronome-based methodology to monitor the stroke length changes in trained swimmers

**DOI:** 10.3389/fspor.2023.1268146

**Published:** 2023-10-17

**Authors:** Marco Fassone, Ambra Bisio, Luca Puce, Monica Biggio, Filippo Tassara, Emanuela Faelli, Piero Ruggeri, Marco Bove

**Affiliations:** ^1^ Department of Neuroscience, Rehabilitation, Ophthalmology, Genetics and Maternal Child Health, Università Degli Studi di Genova, Genoa, Italy; ^2^ Centro Polifunzionale di Scienze Motorie, Università Degli Studi di Genova, Genoa, Italy; ^3^ Department of Experimental Medicine, Section of Human Physiology, Università Degli Studi di Genova, Genoa, Italy; ^4^ IRCCS Policlinico San Martino, Genoa, Italy

**Keywords:** test, training, swimming performance, pacing, timing

## Abstract

The aim of our study was to develop a methodology that uses the metronome to constrain the swimmers' stroke rate with the aim to monitor changes in stroke length (SL) during two different periods of the season. Thirteen young trained swimmers (15.7 ± 1.7 y) performed three 50 m front crawl time trials during pre-season (PRE) and after 2 months, during the in-season period (IN). They were asked: (I) to swim at their maximum intensity (NO-MET condition); (II) to synchronize their stroke with a metronome beat set to their preferred intra-stroke-interval (ISI) (100% condition, corresponding to 48 ± 0.7 cycles/min); (III) to synchronize their stroke with a metronome beat set at 5% higher than their preferred ISI (95% condition, corresponding to 51 ± 0.8 cycles/min). The outcome parameters used to evaluate the performance were ISI, SL and total time of 50 m (TT). In NO-MET condition, results showed that TT in IN improved with respect to PRE, but no changes in ISI and SL. In 100% condition, no differences were obtained between the imposed and the performed ISI, whilst in 95% condition, the performed ISI was lower than the metronome ISI, and lower than that in 100% condition. At last, when using the metronome, SL was higher during IN compared to PRE and SL was lower in the 95% condition compared to the 100% condition. Results indicate that the use of the metronome successfully allowed monitoring changes in SL during different periods of the season. This methodology provides valuable information to coaches and athletes to enhance their performance throughout the season.

## 1. Introduction

In the last years, swimmers and coaches adopted a scientific approach both to plan the competitive season and monitor the training sessions. In this context, scientific research has investigated different methodologies to monitor workload to evaluate physical effort and to collect data on the athlete's response to a specific training program (1). In swimming competition, the objective is to cover a given distance as fast as possible (2). Therefore, average swimming speed, defined by Craig et al. (3) as the product of the stroke length and the stroke rate, is considered one of the most representative measure to improve swimming performance (4). A number of studies showed that an inverse relationship exists between these two parameters namely an increase in stroke rate corresponds to a decrease in SL (3–8). Moreover, stroke rate and SL have been demonstrated to play a key role in monitoring the performance in front crawl competitions (8–11).

The search for the best performance and training has led to a rapid development of technology as support during athletic preparation. Thanks to specific devices, coaches are able to measure, evaluate and control the physiological and technical parameters essential to achieve the sport targets (12–16). Inertial sensors are an example of reliable tool used to monitor stroke rate and SL (14, 17, 18) as well as motion capture system based on video acquisition (19, 20) and infrared technology (21, 22). However, these systems are expensive and require specific expertise both during the acquisition phase and in data analysis. A recent survey of 298 coaches' swimmers (245 males, 53 females) from the United States identified ease of use, accessibility and easy of understanding data as the main characteristics for a monitoring tool while reported the lack of finances, time restrictions and accessibility to suitable testing equipment as the main limitations (23, 24). A device that includes all the above-mentioned requirements is the metronome which provides an instant audio feedback through which athletes modulate their stroke rate (7, 25, 26). Indeed, the metronome is an electronic training device that beeps to help to develop a consistent pace. The device is a cheap, small, waterproof, floatable, and easy wearable tool that can be positioned under the swimming cap. Furthermore, it can be used by athletes and coaches also in open water where, due to the environment, it is more difficult to keep pace compared to the swimming pool (27). Since, as suggested by the scientific literature (23, 28), coaches' perception about the training and evaluation methodology is crucial, this method might answer coaches’ needs and will allow them to efficiently plan the training season. At last, human tendency to synchronize with an acoustic stimulation could explain the potential benefits of using acoustic rhythmical cues during physical practice and sport due to the existence of a central pattern generator in the nervous system, which may serve to regulate temporal functioning and govern rhythm response (29–31).

In the last few years, although some criticisms related to the higher physiological stress and distraction have been evidenced in using the metronome during swimming (25, 26), an increasing number of studies used it during training to optimize stroke rate with the aim to customize the best race strategy (25). Some of them have used metronome to pace the swimming speed and the device emits a sound at designated time intervals, equivalent to the end of each lap (32–35). Other studies have used metronome to evaluate inter-limb coordination (26, 36–38). Another way in which the metronome can be used is as a tool to constrain stroke rate by imposing an intra-stroke-interval (ISI), as already proposed by Alberty and colleagues (26) and Silva and colleagues (7).

The aim of the study was to test the possibility to use the metronome to constrain the inter-stroke-interval to measure changes in stroke length during two different periods of the training season. We hypothesize that, only when using the metronome, changes in SL will be evident, whilst when swimming without metronome this will be not possible.

## 2. Material and methods

### 2.1. Participants

An *a-priori* power analysis was conducted using G*Power (version 3.1.9.7) (39) to determine the minimum sample size required to test the study hypothesis. The effect size was set at 0.25 considered to be medium using Cohen's criteria (40). A F-test considering one group assessing the interaction between PERIOD (*n* = 2) and CONDITION (*n* = 2) was applied with a significance criterion of *α* = 0.05 and power = 0.8. The minimum sample size needed was *N* = 11 for detecting differences in discrimination sensitivity among measurements. Thirteen swimmers from the same team (7 males and 6 females; mean ± standard deviation age 15.7 ± 1.7 years; body mass 58.9 ± 6.9 kg; height 171.1 ± 7.6 cm) volunteered to participate in this study. According to McKay et al. (41) these athletes can be classified as trained/developmental level. They had a competitive swim experience of 7.4 ± 1.6 years, a swimming training frequency of 5 ± 1 session/wk with a total of 10.0 ± 2 h, with a swimming training volume of 30,000 ± 6,000 m/wk during the current season. The training planning includes both high-intensity and high-volume sessions for all participants. Personal-best time in the 50 m freestyle event was 27.33 ± 1.22 s, which corresponds to 536.8 ± 64.0 FINA Points, performance level 4 (42). The study was carried out in accordance with the code of ethics of the World Medical Association (Declaration of Helsinki 2014) for experiments involving humans. The project was approved by the local ethics committee (Comitato Etico per la Ricerca di Ateneo, University of Genova, Italy. N. 2020/21) and written consent was obtained from participants' parents.

### 2.2. Experimental design

The experimental protocol was conducted during pre-season (PRE) and 2 months later, i.e., the in-season period (IN), in a 50 m indoor swimming pool with water temperature at 27 °C. During the 2 months between PRE and IN, swimmers followed their regular training. In both periods, swimmers performed three 50 m front crawl trials with a push-off start and 6 min of rest between trials to attempt total recovery (43). Before each test, swimmers performed a moderate intensity 1,000 m swim warm-up (44).

**Figure 1:**
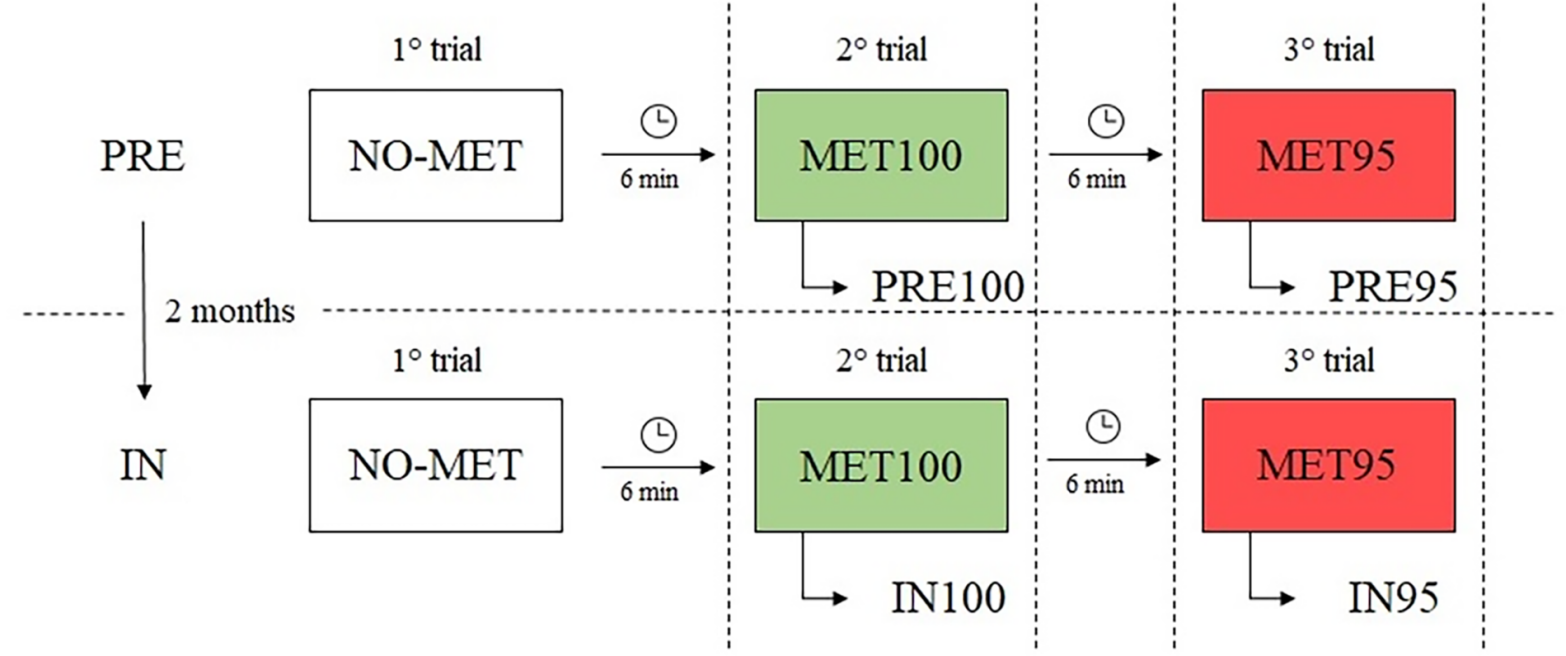
Experimental protocol. Athletes swim 3 times 50 m front crawl during pre-season (PRE) and after 2 months, during in-season (IN). During the first trial swimming was performed without using the metronome (NO-MET, white boxes). The second trial was performed with the metronome set to the individual mean intra-stroke-interval measured in NO-MET (MET100, green boxes). The third trial was performed with metronome set to 95% of the individual intra-stroke-interval (MET95, red box). PRE100 and PRE95 correspond to data collected during PRE at MET100 and MET95, respectively. IN100 and IN95 correspond to data collected during IN at MET100 and MET95, respectively.

In the first trial, participants were asked to swim at their maximum intensity, namely as fast as possible, without the use of metronome (NO-MET condition). The intra-stroke-interval (ISI) was considered as the time between the head emerged from the water (thus excluding the underwater phase) until the end of the lap, divided by the number of strokes. Since ISI quantifies the duration of a single stroke, it also indirectly gives a measure for stroke rate. ISI values of each subject were used as individual reference value to set the interval between two consecutive metronome beats in two conditions: in 100% condition the metronome was set at the mean ISI value corresponding to MET100, whilst in 95% condition the metronome was set at 95% of ISI (MET95), thus imposing a faster stroke rate. In the second trial, participants were asked to swim in synchronous with the audio feedback provided by a waterproof metronome (Tempo Trainer Pro, Finis) positioned under their swimming cap and set at MET100. In the text we will use the acronyms PRE100 when referring to swimmers' data acquired in pre-season, and IN100 when referring to those acquired in in-season. In the third trial, the metronome was set at MET95. In the text we will use the acronyms PRE95 when referring to data acquired in pre-season, and IN95 when referring to IN. In IN, MET100 and MET95 were the same as those imposed in in-season (Figure 1).

### 2.3. Data analysis

Outcome measures used to evaluate swimming performance were: the mean ISI value (s), and the 50 m total time (TT, s; manually recorded by a stopwatch 3X300M Stopwatch, Finis) (45). Furthermore, the meters covered during underwater phase (m), defined as the distance between the wall and the point where the head breaks the surface, evaluated by means of a video camera—GoProR HERO5- at 120 fps and with a resolution of 720 pixels, located at the starting side of pool was used (46) data were analysed with Kinovea, were assessed. At last, the stroke count (SC) were used to compute the stroke length (SL, m) using the following equation: [lap distance (50 m)—underwater phase (m)] / SC. We referred to the work of Girold and colleagues, who used the number of stroke cycles to compute the stroke length (47).

### 2.4. Statistical analysis

Normality was checked by means of the Shapiro-Wilk test. All variables were normally-distributed. Normally distributed data are reported as mean values ± standard error (SE). In NO-MET condition, TT, SL and ISI acquired in PRE and IN periods were compared by means of paired t-test. With the aim to test if participants swam synchronously with the metronome in the two periods of the season and at the two conditions, we applied on ISI values recorded in 100% condition an ANOVA comparing MET100 (namely, the period imposed by the metronome) with PRE100 and IN100 (namely, swimmers' ISI values recorded during the trials), and a second ANOVA on ISI values acquired at 95% condition comparing MET95 with PRE95 and IN95. With the aim to compare ISI and SL when using the metronome in the two periods of the season, an ANOVA with CONDITION (2 levels, 100% and 95%) and PERIOD (2 levels, PRE and IN) as within subject factors was applied. Newman–Keuls *post hoc* tests were applied in case of significant interaction. Hedges' g and partial *η*^2^ values were used to measure the effect size when applying *t*-test and ANOVA, respectively. Concerning Hedges' g, 0.2, 0.5 and 0.8 represent small, medium and large effect, respectively. Concerning partial *η*^2^, 0.01, 0.06, and 0.14 indicated small, medium, or large effects, respectively (48). Linear association between SL and ISI were tested by means of Pearson's correlation coefficient (r) analysis. Data from 100% and 95% condition were pooled together. The level of statistical significance was set at *p* ≤ .05. Statistical analyses were performed with SPSS20.

## 3. Results

### 3.1. NO-MET condition

The statistical analyses showed an improvement in TT during IN (30.57 ± 0.49 s) with respect to PRE (31.04 ± 0.44 s) [*t*(12)= 2.40, *p* = 0.03, *g* = 0.28]. No changes were observed in ISI [PRE: 0.62 ± 0.01 s; IN: 0.61 ± 0.01 s; *t*(12) =  0.93, *p* = 0.37, *g* = 0.22] and SL [PRE: 1.00 ± 0.02 m; IN: 1.00 ± 0.02 *m*; *t*(12)  = 0.51, *p* = 0.62, *g* = 0.11].

### 3.2. Comparison between ISI imposed by the metronome and participants’ ISI

**Figure 2:**
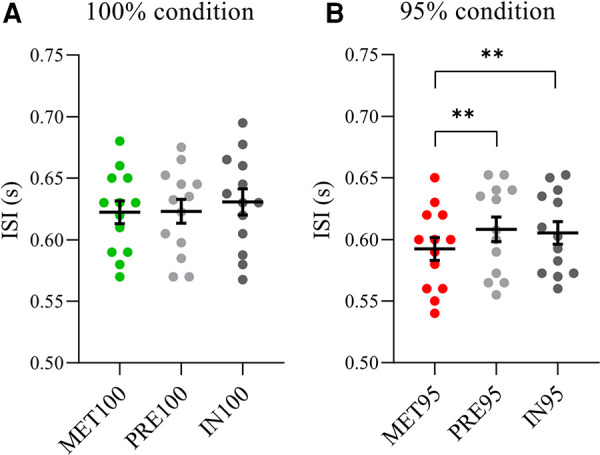
Comparison between ISI imposed by the metronome and participants’ ISI. Intra-stroke-interval (ISI, s) when the metronome was set to the individual time between strokes (100% condition) (**A**) and to its 95% (95% condition) (**B**) Comparison between the metronome values (MET100, green circles or MET95, red circles) and ISI acquired in pre-season (PRE100 during 100% condition; PRE95 during 95% condition, light grey circles) and in-season (IN100 during 100% condition; IN95 during 95% condition, dark grey circles). Horizontal bars represent the mean values and error bars indicate the standard error of the mean. ** *p* < 0.01.

ANOVA on ISI recorded in 100% condition showed no main effect [*F*(2,24)= 2.47, *p* = 0.11, *η*^2 ^= 0.17]. Differently, a main effect on ISI in 95% condition was found [*F*(2,24) = 8.79 *p* = 0.001, *η*^2 ^= 0.42]. Post hoc analysis showed that PRE95 (0.61 ± 0.01 s, *p* = 0.002) and IN95 (0.61 ± 0.01 s, *p* = 0.004) were higher with respect MET95 (0.59 ± 0.01 s) (Figure 2).

### 3.3. Statistical assessment of ISI and Sl in presence of metronome

**Figure 3:**
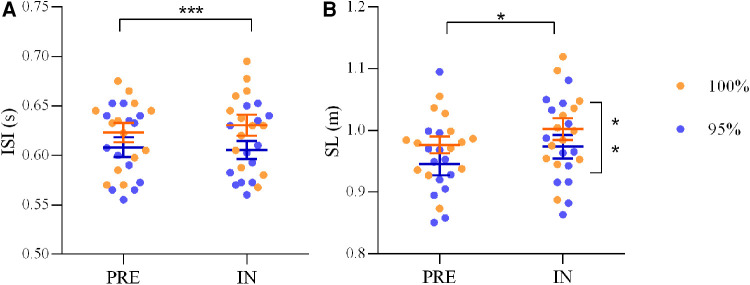
Statistical assessment of ISI and SL in presence of metronome. Comparison between intra-stroke-interval (ISI, s) values (**A**) and stroke length (SL, m) (**B**) in the two period of the season (PRE and IN) during 100% condition (orange circles) and 95% condition (blues circles). * *p* < 0.05, ** *p* < 0.01, *** *p* < 0.001.

**Figure 4:**
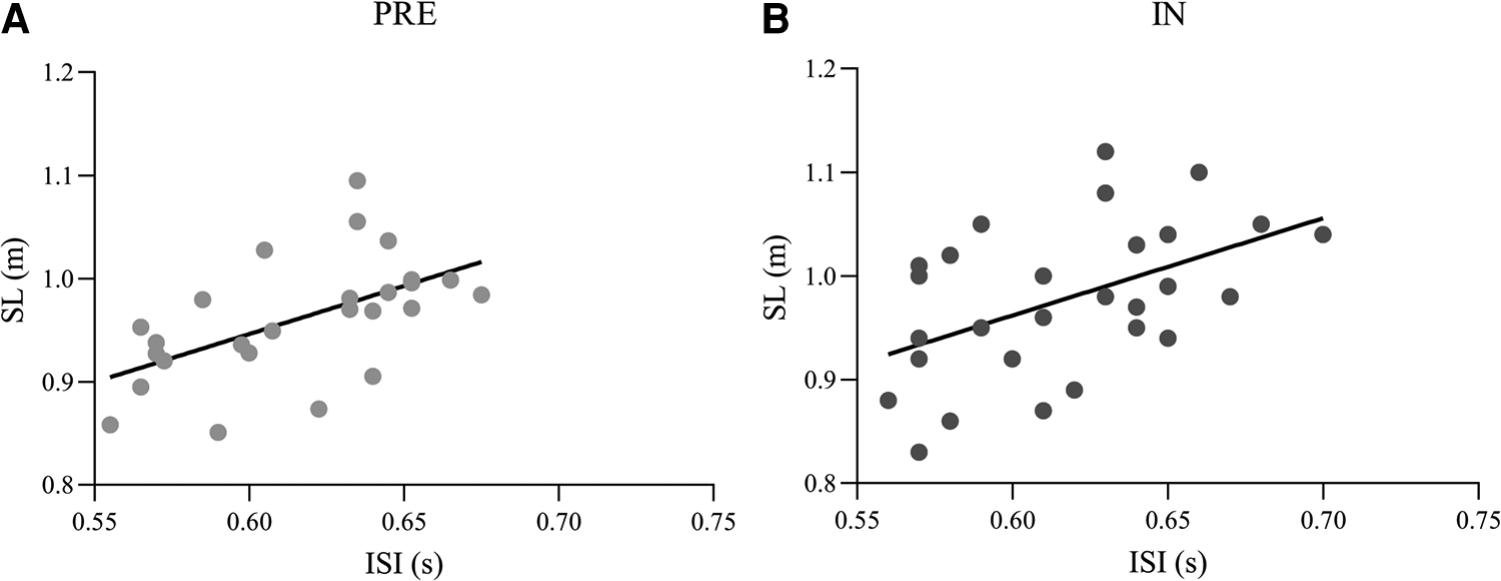
Linear relationship between intra-stroke-interval (ISI) and stroke length (SL) in pre-season (PRE) (**A**) and in-season (iN) (**B**).

ANOVA on ISI highlighted a main effect of CONDITION [*F*(1,12) = 41.61, *p* < 0.001, *η*^2 ^= 0.78] and indicated a lower ISI value in 95% (0.61 ± 0.01 s) with respect to 100% (0.63 ± 0.01 s). No main effect of PERIOD was observed [F(1,12) = 0.55, *p* = 0.47, *η*^2 ^= 0.04]. ANOVA on SL showed a main effect of CONDITION [*F*(1,12) =  12.50 *p* = 0.004, *η*^2 ^= 0.51] that indicated a lower SL value in 95% (0.96 ± 0.07 m) with respect to 100% (0.99 ± 0.06 m). A main effect of PERIOD [*F*(1,12) = 7.71, *p* = 0.017, *η*^2 ^= 0.39], with SL larger in IN (0.99 ± 0.06 m) than in PRE (0.96 ± 0.06 m) was observed (Figure 3). Positive linear relationships between ISI and SL were found in PRE (*r* = 0.56, *p* = 0.003) and IN (*r* = 0.44, *p* = 0.025) (Figure 4).

## 4. Discussion

Results of this study showed that, in NO-MET condition, TT improved during the IN period with respect to PRE period with no changes in SL and ISI. When the metronome was imposed, participants were able to swim in synchronous with the time set by the device only during the 100% condition (imposed stroke rate 48 ± 0.7 cycles/min). Although during 95% condition (imposed stroke rate 51 ± 0.8 cycles/min) ISI was lower than in 100% condition, swimmers were not able to follow the metronome. SL was higher during IN than PRE period and lower during the 95% with respect to the 100% condition. Finally, a linear relationship between ISI and SL in both PRE and IN periods was found.

The beneficial effects of the auditory information on movement control and timing, as well as movement execution, have been shown by several studies (49). Clinical researchers highlighted the use of the metronome to facilitate rehabilitation of intrinsically rhythmical movements including an improvement in gait velocity, step cadence and stride length reducing gait variability (50–55). On the other hand, the use of a specific tool that provides sounds has been shown to favour the optimization of both the movement control and its execution in sports disciplines such as rowing (56), cycling (57) and swimming (58). In swimming research, the metronome was used during training to constrain the stroke rate (6, 7, 26, 59) and the training pacing (32, 34, 35, 60).

In the present study, we proposed a methodology that used the metronome to constrain swimmers' stroke rate and measure the changes in SL in two different periods of the season (PRE and IN) and with two different values of ISI (100% and 95% conditions). Monitoring changes in stroke length is important when considering the results of the studies by Craig et al. (3) and Letzelter et al. (5), who reported that faster swimmers showed longer SL than slower swimmers. Considering the present results, when athletes were free to swim at their own pace, TT improved during IN compared to PRE period suggesting that the regular training performed by the swimmers was effective in improving their performance. However, going deeply into the reasons of this improvement, in NO-MET condition no changes of the parameters describing the performance (i.e., ISI or SL) were found. For this reason, it was not possible to understand what caused the TT improvement. On the other hand, when swimmers were asked to synchronize their strokes with the acoustic stimulus provided by the metronome, it was possible to quantify the changes in SL. The SL was found to be greater in IN period compared to PRE period. Therefore, one might conclude that this methodology has been effective in revealing a change of a parameter crucial for the swimming performance that is not possible to observe in free swimming, except when one uses expensive technologies, challenging to use and not at disposal of all the swimming teams. With respect to these tools, the metronome has the advantage to be a cheap and easy-to-use device, available to all coaches and athletes.

It is worth to note that SL was greater in 100% than in 95% condition. Indeed, when athletes were asked to deviate from their preferred stroke rate, decreasing the ISI value, the stroke efficiency decreased, thus detrimentally influencing the SL. This was also confirmed by the correlations between ISI and SL in both PRE and IN periods, in accordance with a previous study that reported that the improvement in TT was achieved by the combination of increases/decreases in stroke rate and SL (61).

Another point that needs to be discussed is athletes' ability to synchronize their strokes with the acoustic stimulus provided by the metronome. Results showed that they managed to follow the beat of the MET100, namely their preferred stroke rate. Instead, when they were asked to swim at a higher pace, following the beat of the MET95, swimmers tried to speed up the strokes, as demonstrated by the difference between the conditions, but they were unable to follow the metronome. This confirms what has been shown by previous studies where the stroke rate error (error between imposed and preferred stroke rate) was the lowest at the preferred stroke rate (6, 60). Thus, by using the metronome it was possible to unveil that athletes were not able to swim at a rate 5% higher than the own rate. Since previous studies showed a positive relationship between stroke rate and strength (9, 62), this result could be attributed to the need to increment muscle strength. Since the increase in stroke rate is one of the primary factors determining the swimming performance (62), it could be speculated that thanks to this methodology it is possible to quantify how much swimmers can increase the stroke rate, and therefore plan specific training characterized by low-volume and high-velocity/force intensity aimed at improving this performance parameter (63, 64).

## Limitations

A possible limitation of this study is that the proposed methodology was tested only on athletes competing at trained/developmental level. Thus, we are not aware of its effectiveness when applied on athletes of other levels. Future studies to verify the reliability of this metronome-based methodology in other categories are needed. Another limitation is that we neither evaluated their level of maturation nor collected detailed information on the anthropometrics characteristics, except body mass and height. Indeed, both stroke rate and stroke length might be influenced by these aspects.

## Conclusion

In conclusion, the use of the metronome allowed us to quantify the changes of one of the key performance parameters, namely the stroke length. Our findings suggest that coaches and young trained swimmers can monitor with this small, waterproof, easy to set up and economic tool, both the stroke length and the stroke rate during different periods of the training season and then customize their best race strategy. At last, since swimmers were not able to swim with a slower ISI (95% condition), it would be useful to introduce during the in-season training period, resistance exercises characterized by a low-volume and high-velocity/force intensity.

## Data Availability

The raw data supporting the conclusions of this article will be made available by the authors, without undue reservation.
